# Glowing worms: A low-cost fluorescence kit for toxicological education using *Caenorhabditis elegans*

**DOI:** 10.17912/micropub.biology.001506

**Published:** 2025-02-04

**Authors:** Sakina Shahid, Chiara Klein, Joel N. Meyer, Javier Huayta

**Affiliations:** 1 Nicholas School of the Environment, Duke University, Durham, North Carolina, United States

## Abstract

Exposures to metals such as lead and cadmium due to environmental pollution cause cardiovascular, liver, kidney, and other diseases. We used the nematode *Caenorhabditis elegans* paired with an inexpensive “do-it-yourself” fluorescent microscopy setup to highlight the effects of cadmium exposure on expression of the metallothionein-producing gene *mtl-2* to enhance education on the effects of heavy metals. This approach enables observation of GFP fluorescence in worms outside of laboratory settings, while also allowing quantification of gene expression after chemical exposure. We developed an activity centering this experiment for the Museum of Life and Science located in Durham, NC. Participants successfully observed the effects of cadmium on *C. elegans* and made meaningful connections to both environmental and human health.

**Figure 1. Increased fluorescence in CL2122 worms after exposure to cadmium:**
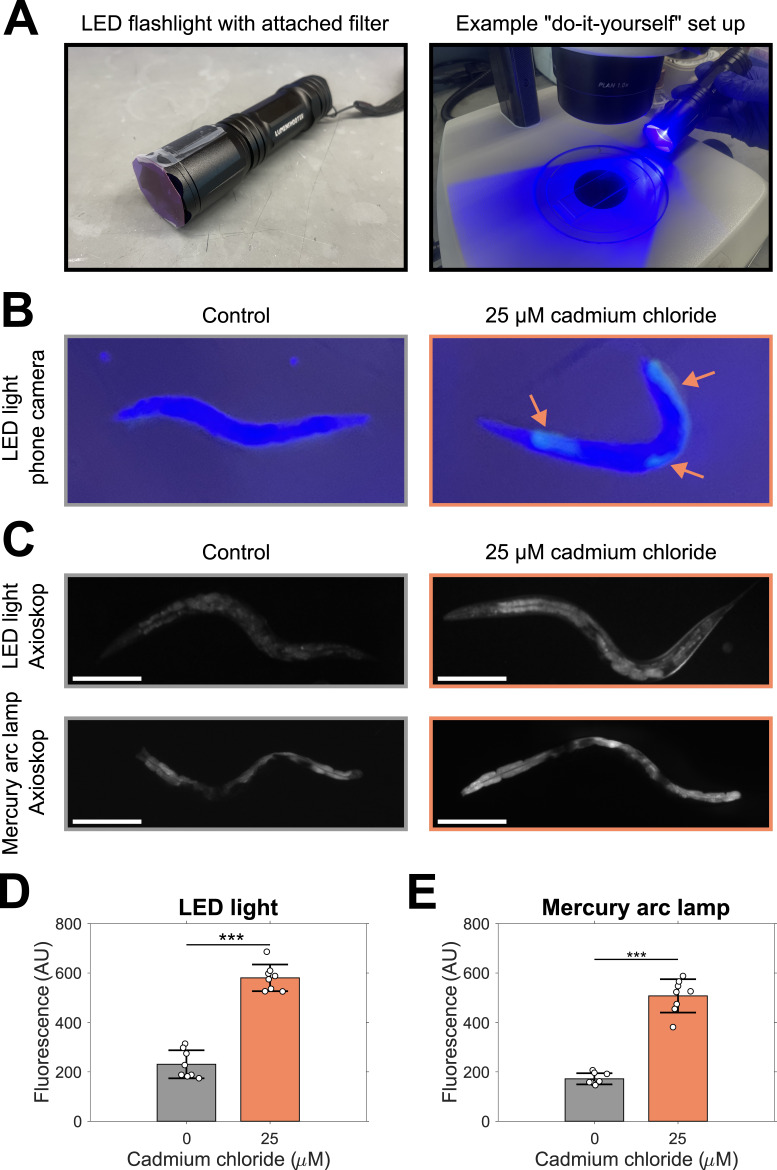
A) LED flashlight with attached lavender plastic filter (left), and example use of LED flashlight for “do-it-yourself” fluorescent microscopy. B) Representative images of control worms or worms exposed to 25 µM cadmium chloride using the LED light and the camera of an Apple iPhone SE; these images were not used for data collection. Red arrows indicate fluorescence visible using the phone camera. C) Representative images of control worms or worms exposed to 25 µM cadmium chloride using the LED light and imaging system of a Zeiss Axioskop (top) or a mercury arc lamp light source and imaging system of a Zeiss Axioskop (bottom). D) Comparison of mean fluorescence between control and cadmium-treated worms using the LED light. E) Comparison of mean fluorescence between control and cadmium-treated worms using a mercury arc lamp light source. Scale bars 200 µM, error bars are standard deviation, n = 8 from one biological replicate, (***) *p* < 0.001 using two-tailed t-test.

## Description

An elevated occurrence of lead (Pb) contamination in urban soils near residential foundations and urban streets compared to background levels was found in suburban areas and city parks in Durham, North Carolina (Wade et al., 2021) . Subsequent research identified sources of lead contamination and reported an increased occurrence of metals such as cadmium and antimony (Wang et al., 2022) . Cadmium has been linked to cardiovascular, liver, and kidney disease in humans (Edwards et al., 2009; Hyder et al., 2013, Tellez-Plaza et al., 2013) , showcasing the importance of educating people on the effects of cadmium in health and its presence in the environment. *C. elegans* is a small nematode that can be found in organic matter at soil surfaces and has been used in toxicological research due to its short lifespan, well-mapped genome, transparent body, and its low maintenance cost compared to other laboratory models (Leung et al. 2008) . In *C. elegans* , the metallothionein-producing gene *mtl-2* is one of many genes that regulate detoxification of the cadmium ion (Hall et al., 2012) . We used a *C. elegans* strain containing gene *mtl-2* tagged with the fluorophore GFP, with expression centered in intestinal cells. Worms exposed to cadmium chloride for 24 hours starting at their L4 larval stage exhibit increased fluorescence due to increased production of the MTL-2 protein in response to the presence of cadmium ion. To enable observation of this phenomenon outside of a laboratory setting, we adapted a previously described method of “do-it-yourself” fluorescence microscopy (Schaefer et al., 2023) . We illuminated the worms with a blue LED light filtered with lavender film plastic (Roscolux R4990 CalColor 90 Lavender), to function as the excitation light ( **Fig. 1A** ). We did not use the optional yellow film plastic for the emission filter as described by Schaefer et al. as it reduced the ability of observers to discern the worms because of increased blurriness. We tested our microscopy setup with untrained observers and found that they were able to see the visible differences between unexposed worms and those exposed to cadmium ( **Fig. 1B** ). Implementation of this system is low cost ( **Table 1** ), facilitating its use for education on the toxicological effects of cadmium.

Afterwards, our team facilitated this experiment at the Museum of Life and Science (Durham, NC) as an activity of the Community Engagement Core of the Duke Superfund Research Center. The activity was designed for a range of age groups, with learning outcomes tailored to participants' existing knowledge and interests. For example, we created a slideshow (available upon request) to highlight key concepts of the activity, including nematodes, cadmium, bioluminescence, the differences between soil and dirt, and a basic introduction to microscopes, allowing for multiple entry points into the activity. In conjunction to the slideshow, we set up three microscopes, one showcasing an untagged worm without any fluorescence, one where the worm had GFP tagging, and one with a tagged worm exposed to cadmium. This setup allowed young children (ages 4-8 years old) to visualize bioluminescence and get introduced to microscopes whereas middle schoolers and high schoolers were able to grasp higher level concepts such as fluorescent-tagging, heavy metals, and using nematodes as indicators of soil health. The purpose of the activity was to present soil as a living organism, display and quantify the impact of an unseen pollutant on soil health in a nematode model, and make broader connections to human health. Several children asked whether the worms would die upon exposure, and if the same could happen to humans. In response, we discussed bioaccumulation and strategies for minimizing exposure to pollutants, while emphasizing the importance of environmental stewardship and protecting ecosystems.

Finally, we confirmed the cadmium-induced expression of *mtl-2* by quantifying the fluorescence intensity induced in the worms using the “do-it-yourself” setup with a Zeiss microscope and camera setup ( **Fig. 1C** ), finding a significant increase in fluorescence in worms treated with cadmium compared to control worms ( **Fig. 1D** ). We repeated these measurements replacing the “do-it-yourself” setup with a Zeiss mercury arc lamp light source and filters, confirming the increase in fluorescent intensity after cadmium exposure ( **Fig. 1E** ).

Here, we have adapted a previously described “do-it-yourself” fluorescent microscopy approach for zebrafish for use in *C. elegans* . Furthermore, we tested this method after exposing worms to cadmium, finding that changes in expression of *mtl-2* ::GFP are measurable using this setup. This educational kit can be further utilized to show the effects of other heavy metals such as lead, copper, zinc, and mercury (Ma et al., 2009; Ye et al. 2010) adding flexibility to showcase additional types of soil contamination.

## Methods

**Strains and maintenance:** *C. elegans* strain CL2122 ( dvIs15 [(pPD30.38) *unc-54* (vector) + (pCL26) *mtl-2* ::GFP] was maintained at 20 °C on K-agar plates seeded with OP50 *E. coli* .

**Chemical exposure:** A population containing day 1-2 adults of *C. elegans* strain CL2122 was treated with sodium hypochlorite solution (10% sodium hypochlorite, 20% sodium hydroxide, 70% water) to recover their eggs. These eggs were transferred to K+ medium for 16 hours and allowed to hatch (Stiernagle et al. 2006) . L1 larval stage worms were moved to K-agar plates seeded with OP50 *E. coli* for 48 hours to allow worms to reach the L4 larval stage. Worms were collected and washed with K-medium three times. Approximately 50-80 worms were put in 24-well plates containing 500 µL K+ medium, HB101 *E. coli* at optical density 2.0, and either 0 or 25 µM cadmium chloride (Sigma-Aldrich) (Williams and Dusenbery, 1988) . After 24 hours, worms were collected and washed with K-medium three times for the fluorescence experiment.

**Fluorescence imaging and quantification:** Control and cadmium-exposed worms were mounted on glass slides containing a 2% agarose pad and covered with a glass coverslip. Glass slides containing worms were then illuminated at a 45-degree angle with a blue LED flashlight (Lumenshooter) that had its light source covered with lavender film plastic (Rosco #4990). Viewers then used either a basic stereoscope or their phones for observation of fluorescence. For fluorescent quantification, glass slides were illuminated as previously described or with a mercury arc lamp (Zeiss). Images were acquired with a CoolSnap FX (Photometrics) camera mounted on an Axioskop (Zeiss) microscope using a 10X objective. Binning 2x2 was used for the Lumenshooter images with no binning used for the arc lamp images. Mean fluorescent intensity was measured using ImageJ. We considered imaging on agar plates, which allow the worms to stay viable longer, but found that they moved too fast for effective fluorescence observations with this set-up. Because the agarose pads eventually dry, desiccating the worms, it is helpful to prepare the slides just before the beginning of the activity.

**Handling and disposal of hazardous chemicals and materials:** To minimize the chances of accidental exposure of the participants to cadmium, we washed the worms three times with K-medium after the end of exposure. We also mounted the worms on glass slides at our laboratory and then transported these to the activity site. We found that worms can survive this way for 3-4 hours, which proved to be enough time for demonstration of the fluorescence activity. After the activity, the glass slides and any other object that had contact with chemicals (e.g. pipette tips) were collected in hazardous waste bags, labeled, and collected by Duke University Occupational and Environmental Safety Office's waste disposal system. LED lights are considered safe and produce very low amounts of UV light. However, to ensure the safety of participants and keep them from directing the LED light to their eyes, only individuals with the proper Laboratory Safety Training (Duke University) were allowed to use and manipulate the LED light during the guided activity.

**Table 1: Starting cost for “do-it-yourself” microscopy setup.**

**Table:** 

**Item**	**Cost (USD)**
*C. elegans* strain CL2122	10
LED flashlight	20-30
Plastic film filter	1-5

**Statistical analysis:** Comparisons were made using the Analysis ToolPak add-in for Microsoft Excel for Office 365.
